# Lipid droplet membrane proteome remodeling parallels ethanol-induced hepatic steatosis and its resolution

**DOI:** 10.1016/j.jlr.2021.100049

**Published:** 2021-02-20

**Authors:** Carol A. Casey, Terrence M. Donohue, Jacy L. Kubik, Vikas Kumar, Michael J. Naldrett, Nicholas T. Woods, Cole P. Frisbie, Mark A. McNiven, Paul G. Thomes

**Affiliations:** 1 VA-Nebraska-Western Iowa Health Care System, Department of Veterans' Affairs, Omaha, NE, USA; 2 Department of Internal Medicine, University of Nebraska Medical Center, Omaha, NE, USA; 3 Department of Biochemistry and Molecular Biology, University of Nebraska Medical Center, Omaha, NE, USA; 4 Department of Genetics Cell Biology and Anatomy, University of Nebraska Medical Center, Omaha, NE, USA; 5 Mass Spectrometry and Proteomics Core Facility, University of Nebraska Medical Center, Omaha, NE, USA; 6 Nebraska Center for Biotechnology, University of Nebraska-Lincoln, NE, USA; 7 Eppley Institute, University of Nebraska Medical Center, Omaha, NE, USA; 8 Department of Biochemistry and Molecular Biology, Mayo Clinic College of Medicine, Rochester, MN, USA

**Keywords:** Ethanol, steatosis, lipid droplet, fasting, proteomics, liver, MS, immunohistochemistry, perilipins, mitochondria, ACSM1, acyl-coenzyme A synthetase 1, ACSM5, acyl-coenzyme A synthetase 5, ATP6V0A1, ATPase 116 kDa subunit a isoform 1, CIDEB, cell death–inducing DFFA-like effector B, COXIV, cytochrome c oxidase subunit IV, DHCR7, 7-dehydrocholesterol reductase, FDR, false discovery rate, G0s2, G0/G1 switch protein 2, HSC70, heat shock cognate protein 70, HSD17β11, hydroxysteroid 17β-dehydrogenase 11, HSD17β13, hydroxysteroid 17β-dehydrogenase 13, HSD17β7, hydroxysteroid 17β-dehydrogenase 7, IPA, ingenuity pathway analysis, LAL, lysosomal acid lipase, LAMP, lysosome-associated membrane protein, LD, lipid droplet, LSS, lanosterol synthase, pATGL, phosphorylated adipocyte TG lipase, PCA, principal component analyses, pHSL, phosphorylated hormone-sensitive lipase, PLIN, perilipin, TE, Tris/EDTA, TFEB, transcription factor EB, TM7SF2, delta (14)-sterol reductase, WB, Western blot

## Abstract

Lipid droplets (LDs) are composed of neutral lipids enclosed in a phospholipid monolayer, which harbors membrane-associated proteins that regulate LD functions. Despite the crucial role of LDs in lipid metabolism, remodeling of LD protein composition in disease contexts, such as steatosis, remains poorly understood. We hypothesized that chronic ethanol consumption, subsequent abstinence from ethanol, or fasting differentially affects the LD membrane proteome content and that these changes influence how LDs interact with other intracellular organelles. Here, male Wistar rats were pair-fed liquid control or ethanol diets for 6 weeks, and then, randomly chosen animals from both groups were either refed a control diet for 7 days or fasted for 48 h before euthanizing. From all groups, LD membrane proteins from purified liver LDs were analyzed immunochemically and by MS proteomics. Liver LD numbers and sizes were greater in ethanol-fed rats than in pair-fed control, 7-day refed, or fasted rats. Compared with control rats, ethanol feeding markedly altered the LD membrane proteome, enriching LD structural perilipins and proteins involved in lipid biosynthesis, while lowering LD lipase levels. Ethanol feeding also lowered LD-associated mitochondrial and lysosomal proteins. In 7-day refed (i.e., ethanol-abstained) or fasted-ethanol-fed rats, we detected distinct remodeling of the LD proteome, as judged by lower levels of lipid biosynthetic proteins, and enhanced LD interaction with mitochondria and lysosomes. Our study reveals evidence of significant remodeling of the LD membrane proteome that regulates ethanol-induced steatosis, its resolution after withdrawal and abstinence, and changes in LD interactions with other intracellular organelles.

Fatty liver (steatosis) is the earliest response by the liver to heavy alcohol (ethanol) consumption (1). However, steatosis is reversible after abstinence from ethanol (2). During continuous (chronic) alcohol (ab)use, fat/lipid that accumulates in the liver reacts with free radicals and other secondary ethanol metabolites generated during ethanol metabolism. These eventually trigger subsequent reactions that damage other biomolecules and organelles and disrupting their biogenesis (3). Thus, with continued heavy drinking, hepatic steatosis can worsen liver damage that progresses to hepatitis, fibrosis, and cirrhosis (1).

Ethanol-induced fatty liver is a consequence of dysregulation, induced by ethanol, of several cellular mechanisms (1). Hepatic ethanol metabolism lowers the hepatic NAD^+^/NADH ratio, which initiates significant metabolic shifts toward reductive synthesis to accelerate the synthesis and slow the oxidation of fatty acids (3, 4). Ethanol induces de novo lipogenesis by activating sterol regulatory element binding protein 1c, carbohydrate response element binding protein, and early growth response-1, each of which stimulates transcription of specific genes involved in lipid biosynthesis (4). Hepatic ethanol oxidation simultaneously disrupts fatty acid oxidation by downregulating the PPAR-α, a transcription factor that activates genes involved in fatty acid oxidation (1). The end result of these changes is hepatic steatosis. The latter is exacerbated by enhanced uptake of serum fatty acids generated by ethanol-induced acceleration of adipose tissue lipolysis and deceleration of hepatic lipolysis and lipophagy, both pathways of lipid droplet (LD) catabolism (2).

LDs in liver cells appear histologically as unstained, sharply defined cytoplasmic vacuoles, which contain neutral lipids enclosed by a single phospholipid membrane that harbors integral and peripheral proteins (5). Those proteins are synthesized on either the endoplasmic reticulum (ER) membrane or recruited directly from the cytosol to the LD membrane (6). All these proteins collectively form the LD membrane proteome, which is represented by enzymes of lipid metabolism, the perilipin (PLIN) family, membrane trafficking proteins, and proteins involved in degradation pathways that regulate distinct LD functions. These include enzymes that liberate fatty acids for energy, membrane biosynthesis, lipid signaling, and sequestration and re-esterification of fatty acids into triglycerides (TGs) (6).Other LD membrane proteins have key roles in creating protein-based tethering complexes to maintain interorganelle contact sites (6). Although these latter proteins are bona fide LD membrane proteins, others transiently localize to LD membranes, depending on the metabolic state of the cell.

The complement of hepatic LD membrane proteins likely changes after chronic alcohol consumption, as other studies reveal that the LD membrane proteome is altered in livers of rodents fed a high-fat diet (7, 8). Here, we report studies that similarly examined whether the liver LD membrane proteome undergoes remodeling after chronic ethanol feeding. We compared the LDs from livers from rats chronically fed an ethanol diet with hepatic LDs from rats pair-fed a control diet, from ethanol-fed rats abstained from ethanol and then refed a control diet for 7 days, and from control and ethanol-fed rats that were subjected to a 48-h fast. We sought to determine whether these treatments altered the LD membrane proteome and whether such changes correspond to the status of fat accumulation in the liver. Here we report that all these interventions induced dynamic changes in the LD membrane proteome that corresponded to previously reported changes in lipid metabolic processes in the livers of ethanol-fed animals (1, 2, 9, 10, 11). Such changes were partially or wholly reversible after cessation of ethanol consumption or fasting.

## Materials and methods

### Reagents

Antibodies to PLIN-3, LAMP1, LAMP2A, HSC70, cytochrome c oxidase subunit IV (COXIV), phosphorylated adipocyte TG lipase (pATGL), phosphorylated hormone-sensitive lipase (pHSL), and mitochondria isolation and mitochondrial complex I activity assay kits were obtained from Abcam (Cambridge, MA). The lysosome isolation kit was from Invent Biotechnologies (Plymouth, MN). Antibodies to hydroxysteroid 17β-dehydrogenase 11 (HSD17β11), hydroxysteroid 17β-dehydrogenase 13 (HSD17β13), and cell death–inducing DFFA-like effector B (CIDEB) were from were from MyBioSource (San Diego, CA). Anti-PLIN-5 was from Santa Cruz Biotechnology, Inc. (Dallas, TX). We purchased a protease inhibitor cocktail, deubiquitylase inhibitors, and other specialized reagents from Sigma (St. Louis, MO).

### Animal treatments

All protocols were approved by the IACUC at the VA Nebraska, Western Iowa Health Care System Research Service. We followed the eighth edition of the Guidelines for the Use and Care of Laboratory Animals, published by the National Institutes of Health. Male Wistar rats, weighing 175–200 g, purchased from Charles River Laboratories (Portage, MI) were weight-matched and fed control or ethanol-containing Lieber-DeCarli diets for 6 weeks. Then, randomly chosen ethanol-fed rats were gradually weaned from the ethanol diet (to avoid withdrawal symptoms) and fed ethanol-free control diet, as described (2) or they (and pair-fed control animals) were fasted for 48 h. At euthanasia, we collected blood from the axillary vessels of each rat while it was under isoflurane anesthesia. After exsanguination and pneumothorax, the liver of each animal was removed and a portion of the tissue was subjected to mitochondrial and lysosomal isolation according to manufacturer's protocol. For MS proteomics analyses, we used three rats per group. For other measurements, the number of animals is stated in the figure legends.

### Serum analyses

We measured the levels of NEFA in sera, using a colorimetric assay kit from Cell Biolabs, Inc. (San Diego, CA).

### Hepatic TGs

Preweighed frozen liver pieces were subjected to total lipid extraction. The filtered lipid extracts were saponified to quantify TGs using a Thermo DMA reagent (Thermo Electron Inc., Middletown, VA). Results are stated as mg TG (using a triolein standard) and normalized per gram of the liver.

### LD isolation

LDs from crude liver homogenates were purified by gradient centrifugation as described earlier (2) with slight modifications. Briefly, post–nuclear supernatant fractions were obtained by centrifugation (1,000*g* for 10 min) of 20% liver homogenates in 60% sucrose (w/v) in Tris/EDTA (TE) buffer (10 mM Tris HCl, pH 7.4, 1 mM EDTA) containing phosphatase, protease, and deubiquitylase inhibitors. LDs were isolated by subjecting post–nuclear supernatant fractions to discontinuous sucrose gradient ultracentrifugation using a SW-28 rotor (30,000*g* for 30 min) as described (2). The white band (LD fraction) at the top of the gradient was collected and further purified by centrifugation (30,000*g*) for 30 min in TE buffer. LDs obtained from the latter steps were subjected to three additional centrifugations (20,800*g*) for 10 min each, to remove loosely bound and/or contaminating proteins and copurifying membranes (12, 13). Purity of isolated LDs was confirmed by the absence of commonly copurifying membranes by Western blot (WB) (Supplemental Fig. S1), as we described before (14). To concentrate LDs, the clear buffer underlying the LDs white band was removed and the LD fraction was brought up to 200 μl with TE buffer, containing the inhibitors mentioned above.

### Detection of proteins on WBs

LD fractions were subjected to BCA protein quantification. Proteins were separated by electrophoresis under denaturing conditions on SDS-polyacrylamide minigels and transferred onto nitrocellulose membranes as described before (2, 15, 16). To determine equal protein load, the membranes were stained with Ponceau S and protein load quantified by densitometry (Supplemental Fig. S1). We further incubated the membranes overnight with primary antibodies at 4°C. After washing, membranes were incubated with secondary antibodies conjugated to green or red infrared dye for 1 h. Proteins were detected using the Odyssey infrared imaging system. We quantified protein band densities with LI-COR® analysis software. Levels of Plin-2, a major LD membrane protein were comparable in LD fractions from different experimental groups after equal protein load (Supplemental Fig. S1). Thus, Plin-2 was used as the normalizer for protein quantification.

### Sample preparation, LC-MS, and protein quantification and identification

LD proteins were electrophoretically run into the top portion of a 12% acrylamide gel under reducing conditions (15, 16). The gels were then fixed and stained with colloidal Coomassie Brilliant Blue G. The large dark single band of protein near the top of the gel was excised and reduced by incubation with DTT and then alkylated with iodoacetamide before washing with 50 mM ammonium bicarbonate in 50% acetonitrile to remove SDS and stain, before protein digestion with trypsin (1 μg trypsin per 20 μg sample protein) for 16 h at 37°C (15, 16). Peptide spectra were acquired on a Q-Exactive-HF (Thermo Fisher Scientific, Waltham, MA), with an online U3000 RSLCnano Liquid Chromatography system (Thermo Fisher Scientific). MS was conducted in a top 15 data-dependent acquisition mode triggering on peptides with charge states 2 to 5 over the mass range of 375–1,500 m/z. The online peptide separation was carried out by first loading the sample isocratically onto a trapping column (C18 Acclaim™ PepMap™100 C18 0.075 × 20 mm, 3 μm, 100 Å) at 5 μl/min in 1.5% acetonitrile, 0.2% formic acid. After 2.8 min, this was then switched in-line with the nano-column and peptides were separated on a 75 μm × 25 cm peptide CSH™ C18 130A, 1.7 μm resolving nano-column (Waters Corp, Milford, MA) using a linear gradient run at 260 nl/min from 5% B to 32% B over 96 min where A is water and 0.1% formic acid and B is 80% acetonitrile and 0.1% formic acid. Protein identification was performed by searching MS/MS data against the Swiss-Prot *Rattus norvegicus* protein database downloaded on February 13, 2019, using the in-house Mascot 2.6.2 (Matrix Science Ltd., London, UK) search engine. The rat database has 36,159 entries. The search was set up for full tryptic peptides with a maximum of two missed cleavage sites. Acetylation of protein N-termini and oxidized methionine was included as variable modifications, and carbamidomethylation of cysteine was set as a fixed modification. The precursor mass tolerance threshold was set to 10 ppm, and the maximum fragment mass error was 0.02 Da. The significance threshold of the ion score was calculated based on a false discovery rate (FDR) of ≤1%. We calculated the FDR by the decoy fusion method using PEAKS studio software. Label-free quantitative analysis and peak list generation were performed using Progenesis QI-P 4.2 (Nonlinear Dynamics, Newcastle, United Kingdom) to conduct comparative proteomics. We quantified fold changes (increase or decrease) in protein expression induced by ethanol feeding, refeeding control diet, and fasting control and ethanol-fed rats by comparing datasets from each of the aforementioned treatment groups with the dataset of the pair-fed control group. We used 3 biological replicates per group, and each sample was run once for MS proteomics. Statistical analyses were performed using ANOVA and the Benjamini-Hochberg method was used to adjust *P* values for the multiple testing–caused FDR. The adjusted *P* ≤ 0.05 was considered significant.

### Bioinformatic analysis

Ingenuity pathway analysis (IPA) (QIAGEN) was used to identify enriched biological function (BF), canonical pathway categories, and regulatory networks of LD membrane-associated proteins. The Gene Ontology (GO) analysis and network for control LD proteome were constructed using Cytoscape 3.7.0 software with the ClueGo plugin.

### Immunofluorescence studies

Liver sections were fixed in 10% formalin, embedded in paraffin, cut into sections (4 μm), and mounted onto slides. After deparaffinization, tissue sections were incubated with either anti-COXIV and anti-Plin-2 or anti-LAMP1 and anti-Plin2, followed by incubation with secondary antibodies (goat anti-rabbit (Alexa Fluor 555) and goat anti-mouse secondary antibody (Alexa Fluor 488)). All images were obtained with a fluorescence-detecting microscope. Fluorescence intensities of staining and lysosome numbers were quantified in multiple images using NIH ImageJ analyzer software.

### Statistical analysis

Data are expressed as the mean values ± SEM. We determined statistical significance between groups by one-way ANOVA, using a Newman-Keuls post hoc analysis. A *P* value ≤ 0.05 was considered statistically significant.

## Results

### Refeeding or fasting after ethanol withdrawal attenuated hepatic steatosis

**Fig. 1:**
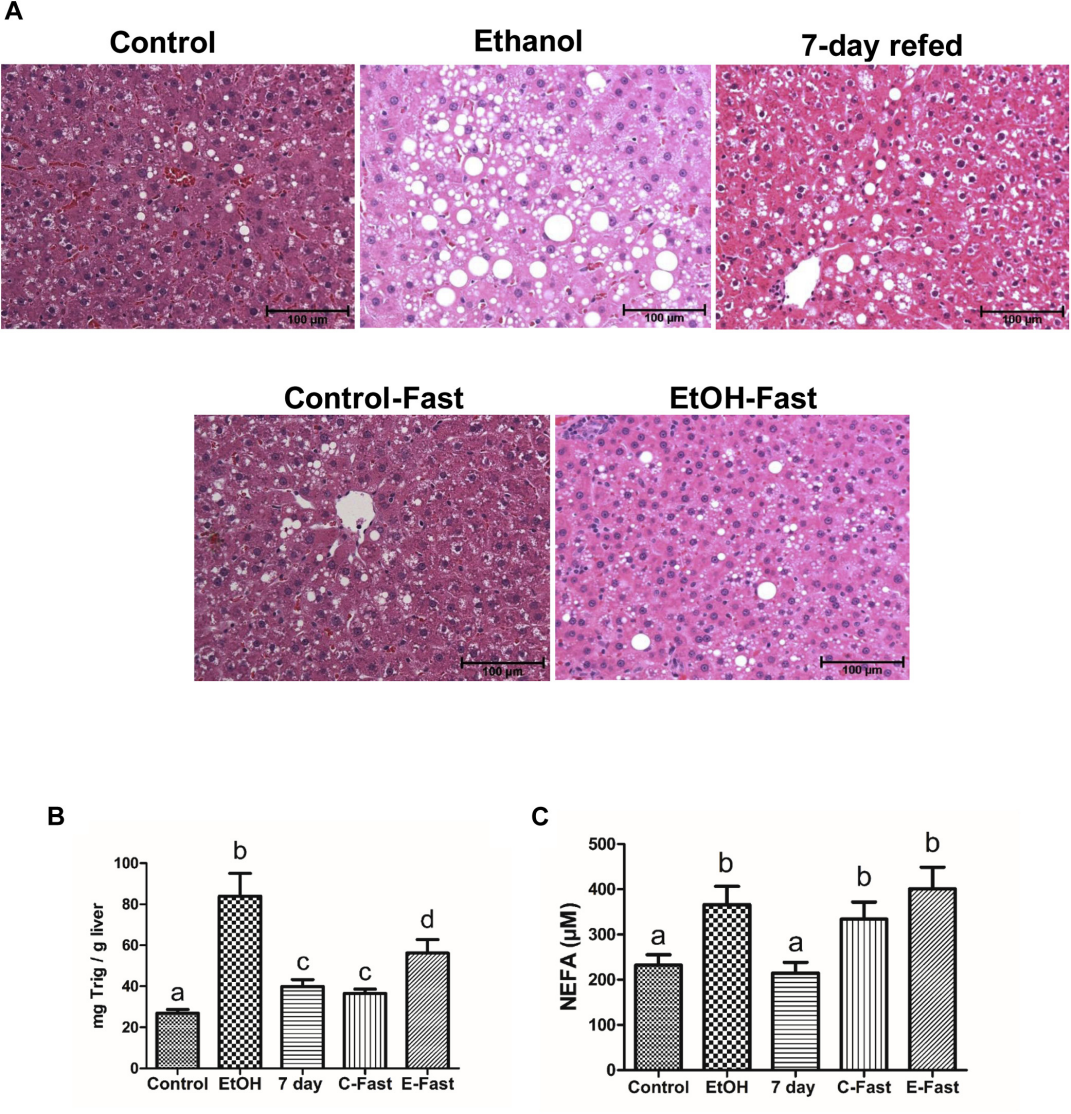
Refeeding and fasting after ethanol withdrawal attenuates hepatic steatosis. A: H&E-stained paraffin section images obtained by light microscopy (B) liver triglycerides and (C) serum NEFA levels from rats treated as indicated in abscissa. Data are the mean values of ± SEM of 6–14 animals per group. Bars sharing different letters are significantly different. Bars sharing the same letter are not significantly different, *P* ≤ 0.05.

We tested whether refeeding ethanol-fed rats the liquid control diet for 7 days (i.e., “7-day refed”) or fasting them for 48 h (i.e., “ethanol-fast”) after ethanol withdrawal attenuates alcohol-induced fatty liver. Liver sections of ethanol-fed rats clearly showed greater numbers of LDs with larger volumes than those of pair-fed controls (Fig. 1A). TG levels in livers of ethanol-fed rats were 3-fold higher than in pair-fed controls and verified the histological findings (Fig. 1B). In sera of ethanol-fed rats, we also detected 1.6-fold higher levels of NEFAs than in pair-fed controls (Fig. 1C). Circulating NEFAs reportedly exacerbate ethanol-induced fatty liver, as they are actively transported into liver cells and re-esterified into TGs (2, 17). In 7-day refed rats, serum NEFAs fell to control levels (Fig. 1C), and while their liver TGs levels declined, they remained significantly higher than in pair-fed controls (Fig. 1B). Compared with rats continuously pair-fed control diet, “control-fasted” rats exhibited 1.4-fold higher levels of both hepatic TGs and serum NEFA levels. Serum NEFA levels in “ethanol-fasted” rats remained unchanged compared with their former ethanol-fed state (Fig. 1B). Liver TGs in these animals remained significantly (1.5-fold) higher than controls.

### Refeeding or fasting after ethanol withdrawal attenuated LD-associated proteins that participate in lipid accumulation and/or LD utilization

**Fig. 2:**
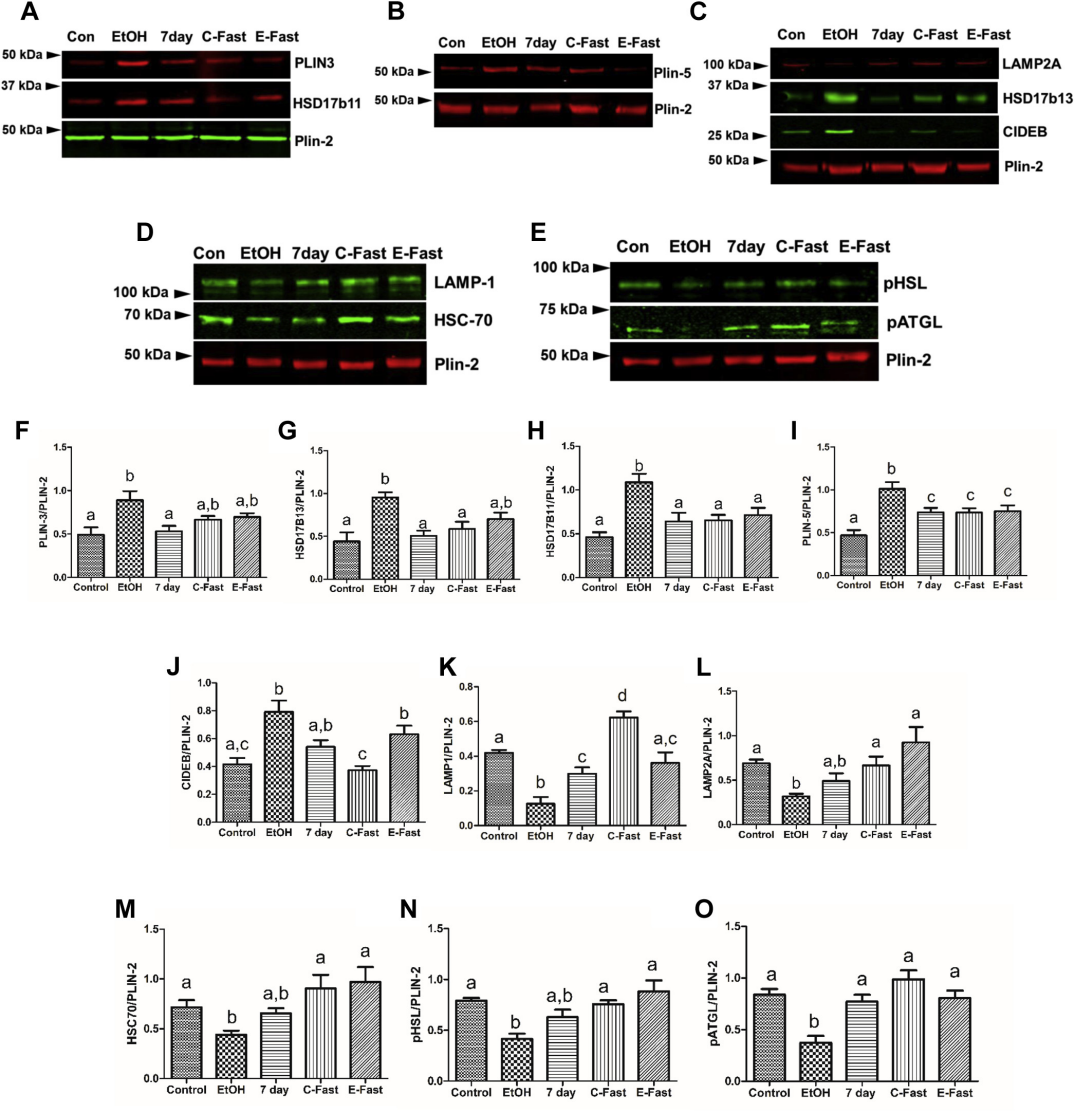
Refeeding and fasting after ethanol withdrawal attenuates LD proteins that participate in lipid accumulation and those that promotes LD utilization. (A–E) Representative Western blots and (F–O) quantification of indicated proteins in lipid droplets isolated from the livers of animals treated as described in the abscissa. Data are the means ± SE of 6–8 animals/group. Bars with different letters are significantly different. Bars with the same letter are not significantly different, *P* ≤ 0.05. LD, lipid droplet.

LD membrane-associated proteins regulate distinct LD functions. Here, using highly enriched (Supplemental Fig. S1) LDs isolated from livers of control, ethanol-fed, 7-day refed, control-fast, and ethanol-fast rats, we measured the contents of selected LD membrane proteins that reportedly regulate LD metabolism. We sought to determine whether expression of these proteins correlates with the status of hepatic fat accumulation. Compared with hepatic LD fractions from pair-fed controls (henceforth, “control(s)”), the levels of the LD membrane proteins Plin-3 and Plin-5 (18) were both 1.8-fold higher in LD fractions from ethanol-fed rats (Fig. 2A, B, F, I). Plin-3 levels in LD fractions of 7-day refed rats returned to control levels, but refeeding caused only a partial decline in Plin-5 levels previously induced by ethanol feeding (Fig. 2A, B, F, I). Plin-5, but not Plin-3, levels were higher in hepatic LDs of control-fast (C-fast) than in LDs of controls, but this Plin-5 level in control-fast animals was 1.4-fold lower than that in LDs of ethanol-fed rats. Both Plin-3 and Plin-5 levels induced by 6 weeks of chronic ethanol feeding were significantly lower in hepatic LDs of “ethanol-fast” rats (Fig. 2A, B, F, I). HSD17β13 and HSD17β11, both implicated in promoting lipogenesis and LD aggregation, respectively (19, 20, 21), were each 2.4-fold higher in LDs of ethanol-fed rats than those from controls (Fig. 2A, C, G, H). Both proteins declined to control levels in LDs from 7-day refed rats, as well as “control-fast” and “ethanol-fast” animals (Fig. 2A, C, G, H). Compared with LD fractions from control rats, the levels of CIDEB, which induces LD fusion (18), causing their enlargement, were ∼1.8-fold higher in LDs of ethanol-fed rats (Fig. 2C, J). CIDEB levels decreased numerically but not significantly after 7-day refeeding and remained comparable with levels in both control and ethanol-fed rats. Compared with controls, “control-fast” and “ethanol-fast” did not change LD-associated CIDEB levels (Fig. 2C, J).

To ascertain LD interaction with other intracellular compartments, we measured the levels of lysosome-associated membrane protein (LAMP) 1 (LAMP1), LAMP2A, and heat shock cognate protein 70 (HSC70). LAMP1 levels indicate intracellular lysosome content (22). LAMP2A and HSC70 coordinate chaperone-mediated autophagy, the lysosomal degradation of signal-bearing target proteins that bind HSC70, a chaperone that mediates their transfer to the lysosome, where lysosome internalization of the target protein is facilitated by LAMP2A. (23). Compared with LDs from controls, LDs from ethanol-fed rats exhibited 3.3-, 2.3-, and 1.5-fold lower levels of LAMP1, LAMP2A, and HSC70, respectively (Fig. 2C, D, K, L, M). Seven-day refeeding after ethanol withdrawal partially restored LAMP1, to near control levels while this treatment and “ethanol-fast” fully restored LAMP2A and HSC70. Compared with LAMP1 in control LDs, “control-fast” significantly elevated LAMP1, whereas LAMP2A and HSC70 were unaffected (Fig. 2C, D, K, L, M). We measured HSL and ATGL, both of which catalyze TG degradation (18). The total content of HSL protein remained unchanged in hepatic LD fractions from all experimental groups. Compared with controls, total ATGL was significantly higher in LDs of ethanol-fed rats but was unchanged in all other groups (Supplemental Fig. S2G, H). Interestingly, however, the active (phosphorylated) forms of both lipases (pHSL and pATGL) were both two-fold lower in LDs of ethanol-fed rats than those of controls (Fig. 2E, N, O). However, after 7-day refeeding, control fast, and ethanol fast, the levels of these phosphorylated lipases in LDs were equal to each other and restored to control levels (Fig. 2E, N, O).

### LDs isolated from control rats revealed LD-associated proteome network

Quantification of selected LD membrane proteins that regulate LD metabolism in livers of all five groups of animals, revealed that, in general, the levels of those proteins correlated with the degree of hepatic LD (fat) accumulation (Figs. 1, 2). To further characterize the dynamic changes in LD membrane-associated proteome that alter LD metabolism, we performed LC-MS analyses to identify and quantify LD membrane proteins from each of the five animal groups. Our LC-MS analyses identified 2,050 LD-associated proteins in control animals (Supplemental Table S1). We compared our dataset with the list of 1,428 LD proteins compiled from different proteomic studies of mammalian cells and tissues, published by Khan *et al.*, (7). Our dataset contained 808 of those 1,428 proteins (Supplemental Table S2). About 150 proteins from our dataset (Supplemental Table S2; highlighted in yellow) that were not found in the study by Khan *et al.*, (7) list, were those recently reported as LD interacting proteins by Krahmer *et al.*, (8), who investigated the hepatic LD–associated proteome in mice challenged with a high-fat diet (8). Our literature review also revealed that many of our proteins that were not found in the list of 1,428 LD proteins mentioned above were found listed as high-confident LD proteins by a study, which used proximity labeling strategy to map the LD proteome (24). The majority of unreported proteins in our dataset belonged to the same protein family (i.e., execute similar BF) that were previously reported. Although our LD preparation is free (14) of some known markers of other organelles (Supplemental Fig. S1), which would suggest that our LD fraction is largely free of contaminating proteins, we acknowledge that sucrose centrifugation methods yield some contaminant proteins, as described in other LD proteomic studies (7, 12, 19, 25).

**Fig. 3:**
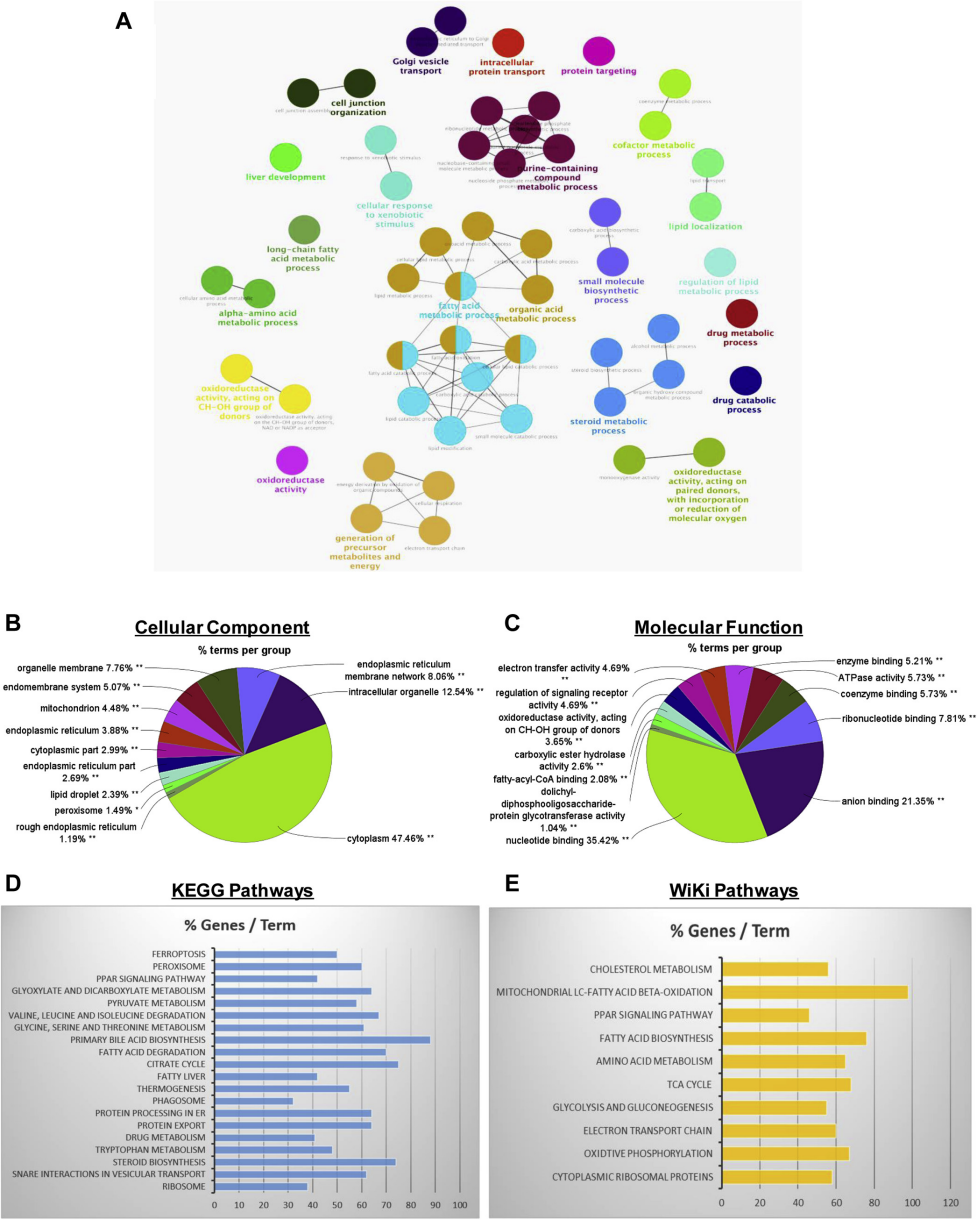
LD-associated proteome network from LDs of control rats. LD proteins are grouped into functional modules based on Gene Ontology (GO) and pathway analysis. A: biological process, (B) cellular component, (C) molecular function, (D) Kyoto Encyclopedia of Genes and Genomes (KEGG) pathway, and (E) WiKi pathway analysis by ClueGo (Cytoscape) of control lipid droplet proteins. LD, lipid droplet.

We next performed GO enrichment analysis with ClueGo Cytoscape plug-in, to determine the functional annotations of our hepatic LD-associated proteome. GO analysis assigned control LD proteome to 58 terms in biological process category (Supplemental Table S3). Figure 3A shows the major biological process categories of the control LD proteome. Among those categories, the top five enriched terms, based on percent of genes/proteins associated with a particular GO term, were fatty acid catabolic processes (30.53%), fatty acid oxidation (27.72%), oxidoreductase activity (26.43%), ER to Golgi vesicle–mediated transport (23.58%), and carboxylic acid catabolic process (22.42%). In the cellular component category, the top five enriched cellular component categories (Fig. 3B) based on % terms per group were cytoplasm (47.46%), intracellular organelle (12.54%), ER membrane network (8.06%), organelle membrane (7.76%), and endomembrane system (5.07%). In the molecular function category, the five enriched molecular function categories (Fig. 3C), based on % terms per group, were nucleotide binding (35.42%), anion binding (21.35%), coenzyme binding (5.73), ATPase activity (5.73%), and electron transfer activity (4.69%). Finally, to determine functionally enriched pathways, we searched both Kyoto Encyclopedia of Genes and Genomes and Wiki databases (Fig. 3D, E). Pathways identified commonly by both pathway analyses were the PPAR signaling pathway, fatty acid metabolic pathway, amino acid metabolic pathway, citrate cycle, and ribosomes. Unique pathways identified by Kyoto Encyclopedia of Genes and Genomes analysis were ferroptosis, peroxisome, bile acid biosynthesis, fatty liver, thermogenesis, phagosome, ER protein processing, protein export, steroid biosynthesis and SNARE interaction in vesicle transport. Unique pathways identified by Wiki analysis were mitochondrial long-chain fatty acid beta oxidation, electron transport chain, and oxidative phosphorylation, all indicating mitochondrial activity.

### Chronic ethanol feeding altered LD membrane-associated proteins to affect their participating pathways

**Fig. 4:**
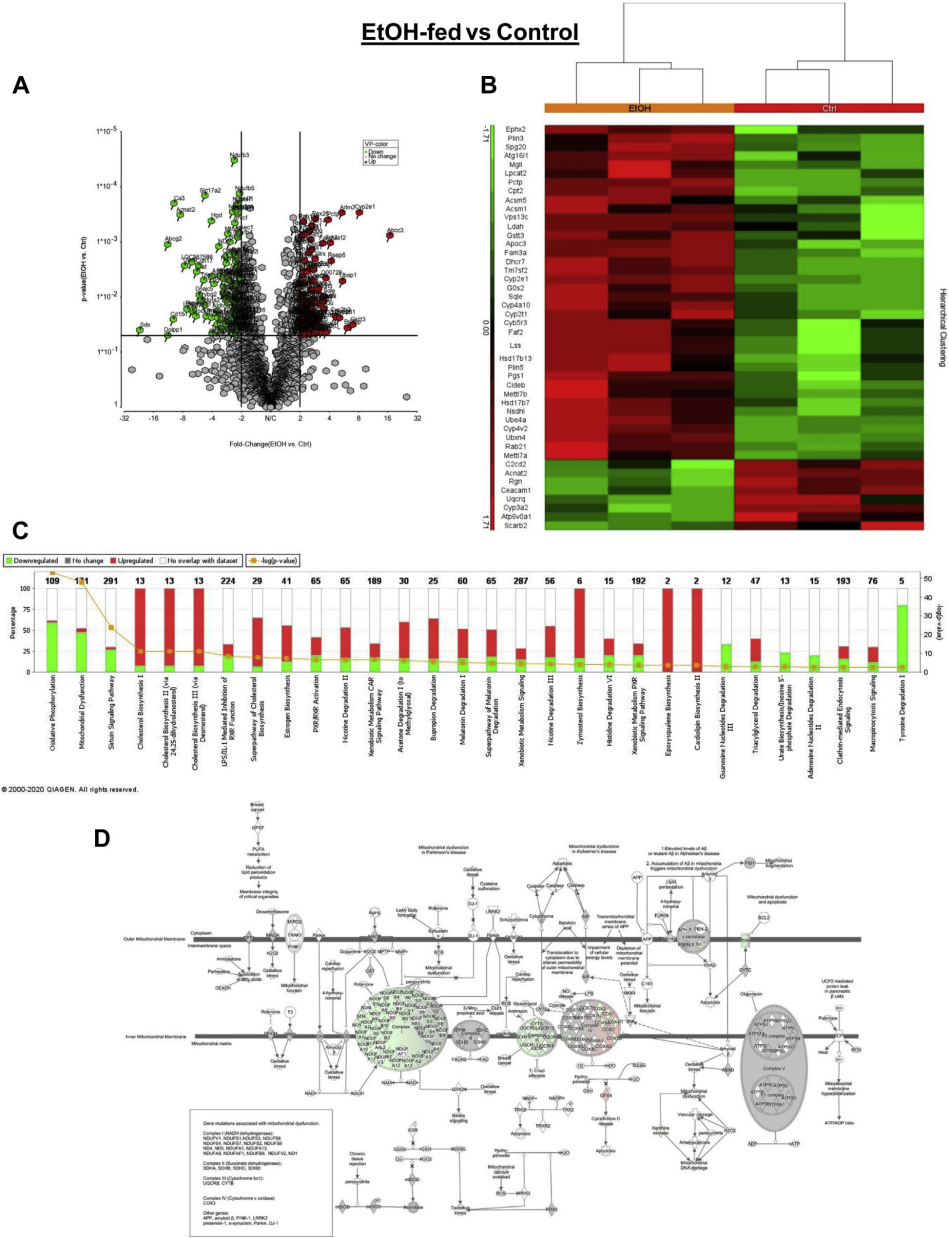
Chronic ethanol feeding altered LD membrane proteins to affect their participating pathways. A: The volcano plot shows the magnitude of fold changes in hepatic LD-associated proteins altered after chronic ethanol consumption by rats. B: The heat map shows changes to frequently reported LD-associated proteins that are induced by chronic ethanol feeding by rats. (C) Top 30 enriched canonical pathways analyzed by Ingenuity Pathway Analysis (IPA) of hepatic LD-associated proteins that are modulated by ethanol. (D) The schematic diagram shows how individual proteins of the top pathway (mitochondria) are affected (in green shade) by chronic ethanol feeding in the rhombus-shaped outline. The volcano plot, IPA, and schematic diagram show significantly (*P* < 0.05) downregulated and upregulated proteins in green and red, respectively (top 30). LD, lipid droplet.

To define ethanol-induced changes to the LD-associated proteome that contributed to hepatic steatosis, we first determined the fold changes in LD membrane proteins induced by ethanol consumption. Compared with controls, ethanol feeding significantly (*P* < 0.05) altered the levels of 338 LD proteins (Fig. 4A and Supplemental Table S4). Among those, 149 proteins, which appear red in the volcano plot (Fig. 4A), were upregulated, whereas 189 proteins, which appear green, were downregulated. The heat map in Fig. 4B shows the status (red: upregulated; green: downregulated) of 48 frequently reported LD-associated proteins. Here, we describe some of the major proteins that reportedly regulate LD metabolism. Plin-3, CIDEB, and HSD17β13, which we found to be upregulated in hepatic LDs isolated from ethanol-fed rats (Fig. 2), were similarly elevated by ethanol feeding (Fig. 4B). Furthermore, downregulation by ethanol of lysosomal integral membrane protein 2 (SCARB-2) and of V-type proton ATPase 116 kDa subunit a isoform 1 (ATP6V0A1), which regulates vesicular acidification (Fig. 4B), provided further evidence of lysosome-LD interaction (Fig. 2). Ethanol feeding also increased the levels of two ATGL inhibitors on LD membranes, the G0/G1 switch protein 2 (G0s2) and Fas-associated factor family member 2 (UBXD8). The former inhibits ATGL TG hydrolase activity (Fig. 4B), whereas the latter enhances the LD size by blocking ATGL activity. Chronic ethanol consumption also suppressed the level of LD-associated carcinoembryonic antigen-related cell adhesion molecule 1, which inhibits fatty acid synthase activity (18). This finding was associated with higher levels of lanosterol synthase (LSS), squalene monooxygenase, and hydroxysteroid 17β-dehydrogenase 7 (HSD17β7), each of which participates in cholesterol biosynthesis (18). Of note, although ethanol feeding downregulated pHSL (Fig. 2E, N), it simultaneously elevated the levels of its partner enzyme monoacylglycerol lipase, which catalyzes monoglyceride hydrolysis (Fig. 4B). Next, using the IPA, we identified the canonical pathways to which proteins, significantly altered by ethanol, were assigned. In Fig. 4C, we show those that appeared among the top 30 enriched pathways. Those proteins that were significantly decreased (% decrease; appear in green) were those involved in oxidative phosphorylation, other mitochondrial pathways and sirtuin pathways (Fig. 4C). Those proteins that were significantly increased (% increases appear in red) were those involved in cholesterol and steroid hormone biosynthesis (Fig. 4C). Because the prominent downregulated pathways all indicated mitochondrial dysfunction, we identified, by IPA, those functions specifically affected. Chronic ethanol feeding decreased the levels (shown in green) of mitochondrial complexes I, III, and IV, predominantly those associated with complex I (Fig. 4D).

### Refeeding (7-day refed) and fasting (ethanol-fast) after ethanol withdrawal caused distinct changes in the LD membrane-associated proteome

**Fig. 5:**
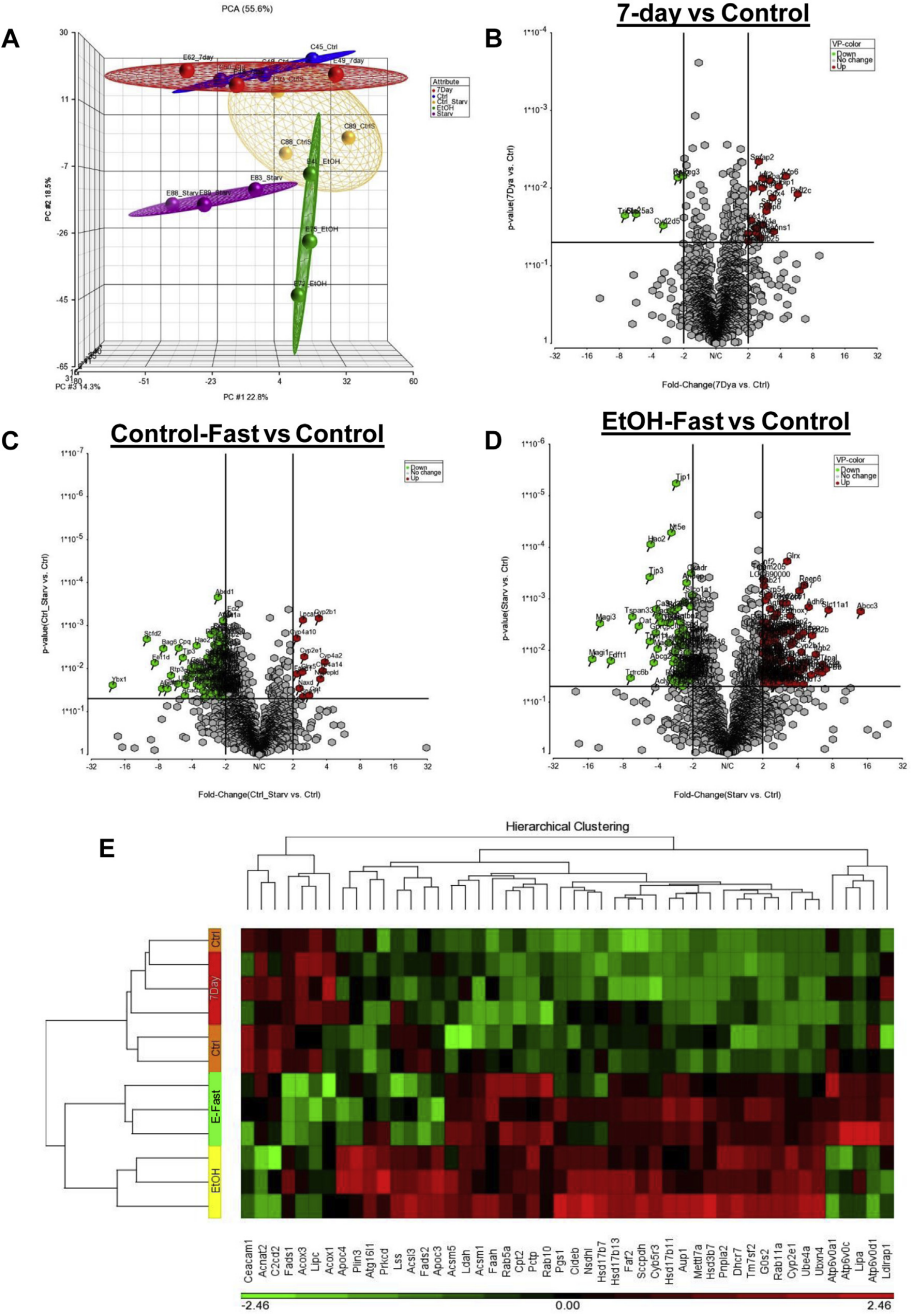
Refeeding and fasting after ethanol withdrawal induced distinctive changes in LD membrane proteome content. A: Principal component analysis (PCA) compares control (blue), ethanol (green), 7-day (red), control-fast (yellow), and EtOH-fast (purple) groups. Volcano plot show magnitude of fold changes of proteins significantly (*P* < 0.05) altered in LDs from (B) 7 days refed (C) control fast, and (D) ethanol fast. E: The heat map show changes induced by ethanol and 7-day refeeding and fasting of ethanol-fed rats after ethanol withdrawal, to selected bona fide and LD-associated lipid-metabolizing proteins. LD, lipid droplet.

To determine LD-associated proteome remodeling associated with resolution of fatty liver by 7-day refeeding or ethanol fast, we performed principal component analyses (PCA) of datasets from LDs of our five animal groups to visualize clustering of samples within each group and overlap among groups. PCA revealed control (blue), ethanol (green), and ethanol-fast (purple) samples were clearly separated from each other with no overlap (Fig. 5A). LDs from 7-day refed rats (red) were separated from ethanol and ethanol-fast but clustered with control samples. Control-fast samples (yellow) showed larger intersample variation and resembled control, 7-day refed, and ethanol-fed samples (Fig. 5A). We next, examined individual LD proteins that were altered by refeeding and ethanol fast. As described in the volcano plot (Fig. 4A), ethanol feeding induced significant changes in the LD-associated proteome. We then compared all other groups to controls and found that 7-day refeeding significantly altered the levels of 93 proteins (Supplemental Table S5). Among them, 68 proteins were upregulated and 28 proteins were downregulated (Fig. 5B). Compared with controls, control-fast rats exhibited 245 proteins that were significantly altered of which 53 proteins were upregulated and 192 proteins were downregulated (Fig. 5C and Supplemental Table S6). Compared with controls, ethanol-fast LDs had significantly altered 448 proteins (Supplemental Table S7), of which 270 were upregulated and 178 were downregulated (Fig. 5D). To determine how changes in individual LD proteins contribute to the status of hepatic fat accumulation, we first generated a heat map (Fig. 5E) with some bona fide LD proteins and with those that regulate lipogenesis and lipid catabolism and are significantly altered by ethanol, 7-day refeeding, and ethanol fast. The heat map is shown in green-red color scale, where the intensities of green and red represent the degree of downregulation and upregulation, respectively. Carcinoembryonic antigen-related cell adhesion molecule 1, Plin-3, CIDEB, HSD17β7, HSD17β13 and G0s2 proteins, each elevated by ethanol feeding (Fig. 4B) were all normalized to control levels in hepatic LDs from 7-day refed and ethanol-fast rats (Fig. 5E). Proteins involved in steroid hormone and cholesterol biosynthesis (26) including LSS, HS17β7, HS17β11, hydroxysteroid 3β-dehydrogenase 7, cytochrome b5 reductase 3, cytochrome P4502E1, sterol-4-alpha-carboxylate 3-dehydrogenase, decarboxylating, delta (14)-sterol reductase (TM7SF2), and 7-dehydrocholesterol reductase (DHCR7) were all upregulated in LDs of ethanol-fed rats but they fell to control levels in 7-day refed animals. In ethanol-fast LDs, except for HS17β11, DHCR7, TM7SF2, and cytochrome P4502E1, which significantly declined compared with ethanol-fed LDs, all other proteins reached control levels (Fig. 5E). Fatty acid activation enzymes acyl-coenzyme A synthetase 5 (ACSM5) and acyl-coenzyme A synthetase 1 (ACSM1) were upregulated in LDs of ethanol-fed and ethanol-fast rats but normalized in LDs of 7-day refed rats. Compared with controls, fatty acid β oxidation proteins, peroxisomal acyl-coenzyme A oxidase 3, peroxisomal acyl-coenzyme A oxidase 1, and hepatic TG lipase were downregulated in LDs of both ethanol-fed and ethanol-fast animals. However, all these proteins returned to control levels in LDs of 7-day refed animals (Fig. 5E). Interestingly, LD acid hydrolase and ATGL (a.k.a. PNPLA2) that break down LDs were elevated in LDs of both ethanol-fed and ethanol-fast rats, but in LDs of 7-day refed animals, these proteins were equal to those of controls. Finally, ATP6V01 and V-type proton ATPase 16 kDa proteolipid subunit, which maintain lysosomal acidification, were all lower than controls in LDs of ethanol-fed rats, but they rose to control levels in LDs from 7-day refed animals. Of note, LDs from ethanol-fast rats exhibited significant upregulation of the latter two proteins compared with LDs from control, ethanol-fed, and 7-day refed rats (Fig. 5E).

### Seven-day refed and ethanol-fast LDs reversed canonical pathways of LD membrane-associated proteins regulated by ethanol

**Fig. 6:**
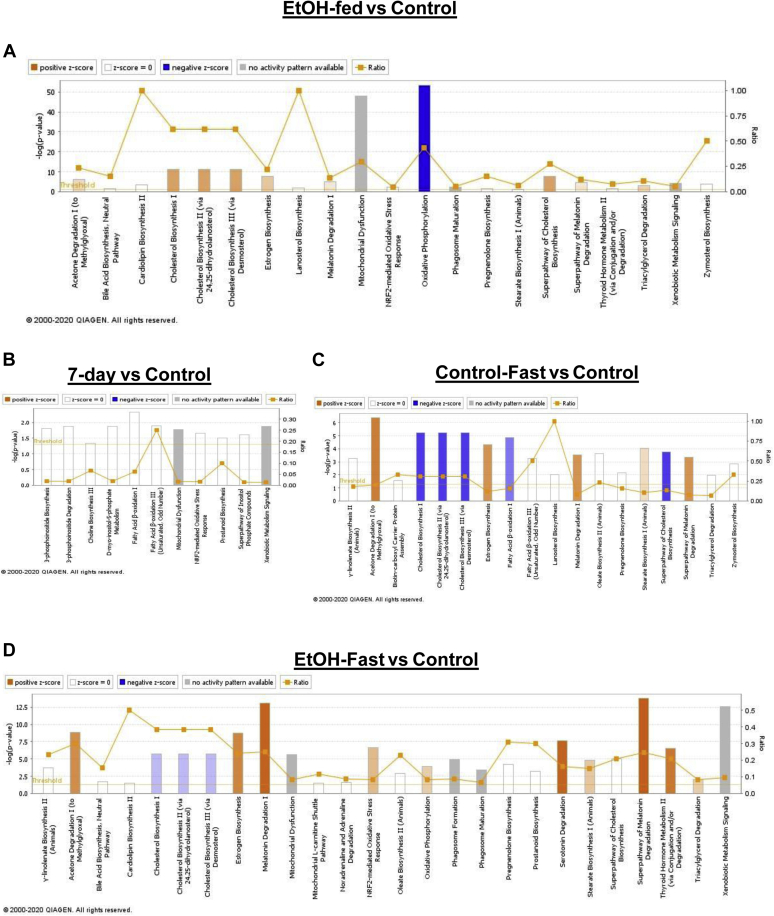
Canonical pathways of LD membrane proteins regulated by ethanol are reversed by refeeding (7-days) and fasting after ethanol withdrawal. Changes induced by (A) ethanol, (B) 7-day refeeding after ethanol withdrawal, (C) control fast, and (D) ethanol fast to commonly identified canonical pathways (among enriched top 25) determined by IPA. Positive Z-score indicates upregulated and negative Z-score indicates downregulated proteins, all versus control, which are shown in orange and blue bars (shades), respectively. White bars indicate no change compared with the control. Gray bars indicate activities not predicted. IPA, ingenuity pathway analysis; LD, lipid droplet.

The heat map shown in Fig. 5E indicates that 7-day refeeding or 48-h fasting of ethanol-fed rats (ethanol-fast) attenuated ethanol-induced elevations of major LD proteins of lipid metabolism. Next, to determine the pathways to which proteins assigned by IPA are affected, we conducted the IPA of all proteins whose levels were significantly altered, from those of controls in all other groups. Pathways identified by IPA are shown in orange, blue, white, and gray bars (Fig. 6). The intensities of orange and blue indicate the degree of upregulation and downregulation, respectively, of the indicated pathway. White bars indicate no significant changes compared with control. The IPA revealed that LD proteins upregulated by ethanol feeding belong predominantly to cholesterol and steroid hormone biosynthetic pathways, whereas LD proteins downregulated by ethanol feeding belong predominantly to oxidative phosphorylation pathways (Fig. 6A). In LDs from 7-day refed animals, no activity score was assigned to many pathways such as cholesterol biosynthesis, nuclear factor erythroid 2-related factor 2-mediated oxidative stress response, and fatty acid beta oxidation, as all these pathways returned to control levels (Fig. 6B). Fasting of control rats (control fast) activated acetone degradation, melatonin degradation, and estrogen and stearate biosynthesis, whereas it downregulated cholesterol biosynthesis and fatty acid oxidation (Fig. 6C). Fasting of ethanol-fed rats (ethanol-fast) activated degradation pathways, for acetone, melatonin, and serotonin and activated the nuclear factor erythroid 2-related factor 2-mediated oxidative stress response and oxidative phosphorylation, but fasting downregulated cholesterol biosynthesis. Because the IPA did not predict the status of mitochondrial function in ethanol-fed, 7-day refed, and ethanol-fast groups, we used LDs to quantify the changes in mitochondrial proteins. Ethanol feeding downregulated 38 distinct proteins that participate in the mitochondrial pathway. LDs from 7-day refed and ethanol-fast animals each showed restored mitochondrial function that was previously suppressed in LDs of ethanol-fed rats (Supplemental Table S8). To confirm the latter proteomic findings, we subjected LD fractions to SDS-PAGE/WB analysis. Ethanol feeding indeed reduced the levels of voltage-dependent anion channel and synaptosomal-associated protein 23 required for LD interaction with the mitochondrion (27, 28, 29) (Supplemental Fig. S2). Both refeeding the control diet to and fasting of ethanol-fed rats restored these proteins to control levels.

### Fasting or refeeding after ethanol withdrawal each promoted lipid utilization and mitochondrial function

**Fig. 7:**
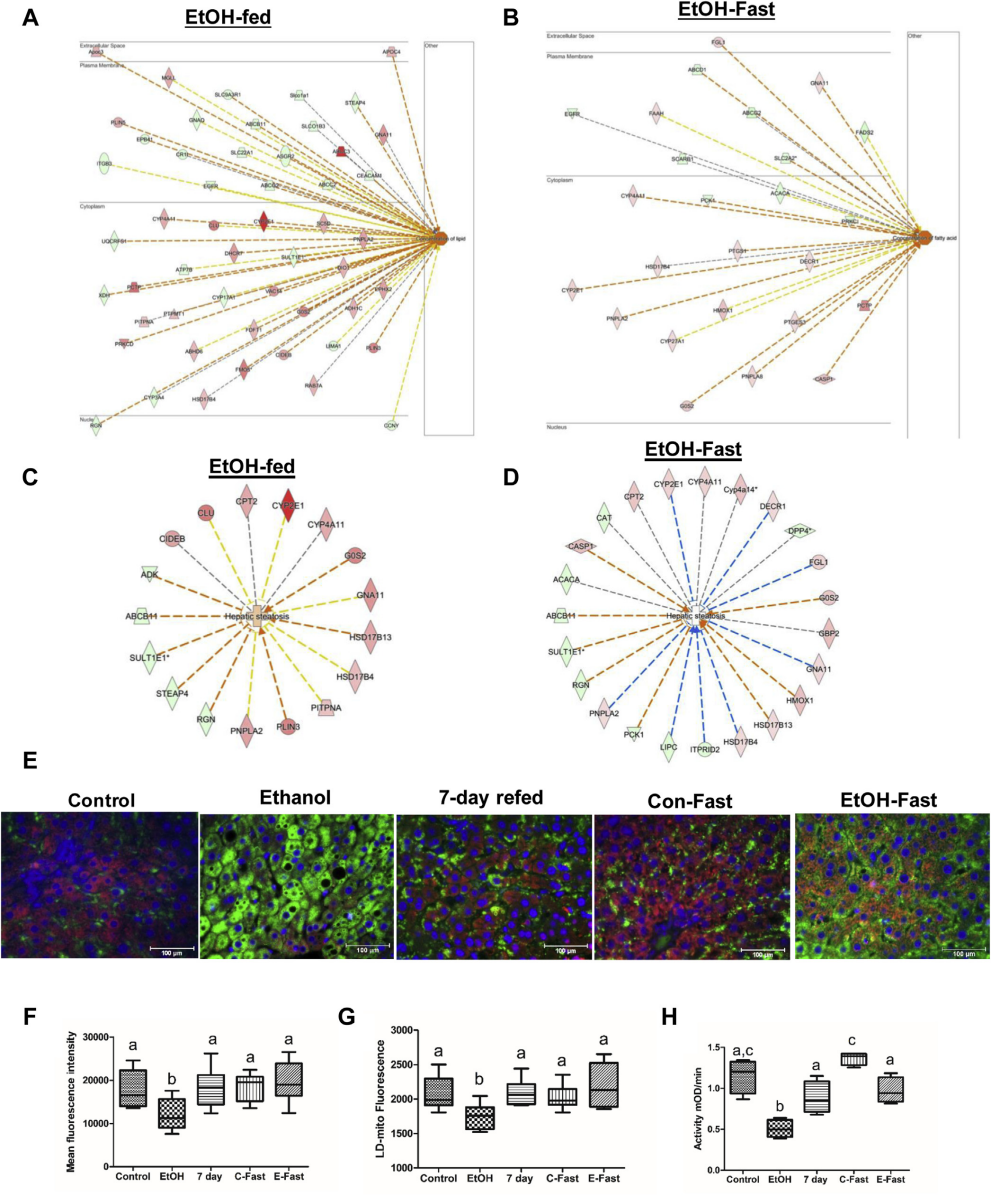
Fasting after ethanol withdrawal reduces lipids and restores mitochondrial function. Network analysis of proteins that contribute to (A and B) lipid concentration and (C and D) hepatic steatosis in LDs of ethanol-fed rats and ethanol-fed rats fasted after ethanol withdrawal by IPA. Upregulated proteins are highlighted in red and downregulated proteins are highlighted in green. The orange dashed line indicates leading to activation while the blue dashed line indicates leading to inhibition of indicated pathway indicated. (D) Immunostaining of liver sections with antibody to mitochondrial marker protein COXIV (red) and lipid droplet marker protein Plin-2 (green), from rats treated, as indicated. Fluorescence intensity quantification by ImageJ of (F) total mitochondrial staining and (G) mitochondrial staining around lipid droplets. (H) Mitochondrial complex I activity in mitochondria isolated from livers of rats treated as indicated on the graph. COXIV, cytochrome c oxidase subunit IV; IPA, ingenuity pathway analysis; LD, lipid droplet; PLIN, perilipin.

Seven-day refeeding of the control diet to or 48-h fasting of ethanol-fed rats each reversed ethanol-induced cholesterol biosynthesis and mitochondrial dysfunction, to suggest that each of these dietary adjustments improved hepatic lipid utilization after ethanol withdrawal. To further examine how 7-day refeeding and ethanol-fast affected lipid metabolism. We reviewed the IPA data analysis for BF categories regulated by these post-ethanol feeding treatments. Compared with controls, none of the BF categories was significantly altered in LDs of 7-day-refed rats (data not shown). However, ethanol feeding modulated (increased or decreased) 43 different LD-associated lipid-metabolizing proteins to elevate intracellular lipids (Table 1, Fig. 7A), but ethanol feeding also caused a decline in LD proteins that carry out carboxylic acid and ion transport (Table 1). In LDs of ethanol-fast rats, activated LD membrane-associated proteins promote: (1) lipid hydroxylation, during which fatty acids are converted to long-chain dicarboxylic acids, preferentially metabolized by the peroxisome beta-oxidation system and subsequently by mitochondria; (2) conjugation of eicosanoids (long-chain polyunsaturated fatty acids), a process that leads to their catabolism; (3) exocytosis; and (4) secretory pathway, both of which are predominantly executed by the Rab family and other vesicle-trafficking proteins (Table 2), which also participate in LD catabolism via lysosome-dependent lipophagy. Of note, the number of proteins (43) that promoted lipid accumulation in LDs of ethanol-fed rats was reduced to less than half (18) in ethanol-fast animals (Tables 1 and 2, Fig. 7B). Ethanol fast simultaneously decreased carboxylic acid transport and fatty acid transport, pathways, largely regulated by related fatty acid trafficking proteins (Table 2). Next, we reviewed the hepatic steatosis functional annotation category and found that ethanol feeding modulated more proteins (orange dashed arrows) that contribute to hepatic steatosis and received a positive Z-score by IPA, indicating upregulation of steatosis (Fig. 7C). The IPA of hepatic steatosis category for ethanol-fasted rats revealed that this treatment modulated more proteins that inhibit (blue dashed arrows) hepatic steatosis. This received a negative Z-score, indicating attenuation of fatty liver (Fig. 7D). Finally, to confirm that mitochondrial function was restored after 7-day refeeding and/or ethanol fast, as observed earlier (Supplemental Table S8), we stained liver tissue from all five groups of animals for mitochondrial COXIV and for Plin-2, and we quantified total mitochondria by their staining around LDs. Compared with livers of control rats, livers of ethanol-fed animals exhibited significantly lower mitochondrial staining overall, as well as around LDs (Fig. 7E–G). However, in livers of 7-day refed and ethanol-fast rats, total mitochondria and their staining around lipid droplets were restored to normal (control) levels (Fig. 7E–G). We then quantified mitochondrial complex I activity in mitochondria-enriched fractions. Compared with controls, EtOH feeding decreased mitochondrial complex I activity by two-fold (Fig. 7H), while complex I activity in enriched fractions from 7-days refed and EtOH-fast rat livers were both equal to controls (Fig. 7H).

**Table 1:** Biological function categories altered by chronic ethanol feeding

Control Versus Ethanol						
Categories	Diseases or Functions Annotation	*P*-Value	Predicted Activation State	Activation Z-Score	Molecules	# Molecules
Lipid metabolism	Concentration of lipids	5.82E-09	Increased	2.273	Abcb11,Abcc2,Abcc3,Abhd6,Apoc4,Asgr2,Atp7b,Ccny,Ceacam1,Cideb,Clu,Cr1l,Cyp17a1,Cyp2e1,Dhcr7,Dio1,Egfr,Epb41,Fdft1,Fmo5,Gna11,Gnaq,Hsd17b4,Itgb3,Lima1,Mgll,Pctp,Pitpna,Plin3,Plin5,Pnpla2,Prkcd,Ptpmt1,Rab7a,Rgn,Sc5d,Slc22a1,Slc9a3r1,Slco1a1,Steap4,Uqcrfs1,Vac14,Xdh	43
Molecular transport	Transport of carboxylic acid	9.49E-09	Decreased	−2.091	Abcb11,Abcc2,Abcc3,Atp7b,Bsg,Cpt2,Prkcd,Slc16a4,Slc23a1,Slc25a1,SlSlc26a1,Slco1a1,Slco1a4	13
Molecular transport	Transport of ion	6.23E-06	Decreased	−2.204	Abcc2,Abcc3,Ano10,Atp2b1,Atp6v0a1,Atp7b,Ca3,Coro1a,Gjb1,Nnt,Nsf,Rab11b,Slc17a2,Slc22a1,Slc23a1,Slc26a1,Slc9a3r1,Slco1a1,Slco1a4,Steap4,Stim1,Vdac2	22

IPA, ingenuity pathway analysis; LD, lipid droplet.

The status of enriched biological function categories of LD-associated proteins altered by ethanol feeding based on Z-scores assigned by the IPA.

**Table 2:** Biological function categories modulated by fasting of ethanol-fed (ethanol-fast) rats

Control Versus Ethanol Fasted						
Categories	Diseases or Functions Annotation	*P*-value	Predicted Activation State	Activation Z-Score	Molecules	# Molecules
Lipid metabolism	Hydroxylation of lipid	5.5E-11	Increased	2.127	Cyb5a,Cyp27a1,Cyp2e1,Cyp4b1,	4
Lipid metabolism	Concentration of fatty acids	7.88E-08	Increased	2	Abcd1,Abcg2,Casp1,Cyp27a1,Cyp2e1,Decr1,Egfr,Faah,Fads2,G0s2,Gna11, sd17b4,Pck1,Pcp,Pnpla2,Ptgs1,Scarb1,Slc2a2	18
Lipid metabolism	Conjugation of eicosanoid	3.83E-07	Increased	2.219	Faah,Ugt1a1,Ugt2b10	3
Lipid metabolism	Conjugation of 12-hydroxyeicosatetraenoic acid	6.21E-06	Increased	2	Ugt1a1,Ugt2b10	2
Cellular function	Exocytosis by cells	1.75E-05	Increased	2.442	Arf6,Napa,Rab21,Rab5a,Rab9a,Rhoa	6
Cellular function	Secretory pathway	6.77E-05	Increased	2.428	Arf6,Napa,Prkcd,Rab11b,Rab21,Rab5a,Rab9a,Rhoa,Sptbn2	9
Cellular function	Exocytosis	0.000125	Increased	2.428	Arf6,Napa,Prkcd,Rab21,Rab5a,Rab9a,Rhoa,Sptbn2	8
Molecular transport	Transport of carboxylic acid	1.96E-12	Decreased	−2.09	Abcb11,Abcc2,Abcc3,Abcc6,Abcd1,Abcd3,Abcg2,Alb,Ca14,Cpt2,Prkcd,Scarb1,Slc16a2,Slc23a1,Slc26a1,Slc27a2,Slc2a2, Slco1a1,Slco1a4	19
Lipid metabolism	Transport of fatty acid	1.16E-09	Decreased	−2.182	Abcc2,Abcc3,Abcc6,Abcd1,Abcd3,Abcg2,Alb,Cpt2,Prkcd,Scarb1,Slc27a2,Slco1a1,Slco1a4	13

IPA, ingenuity pathway analysis; LD, lipid droplet.

Status of enriched biological function categories of LD-associated proteins modulated by fasting of ethanol-fed rats, based on Z-scores assigned by the IPA.

### 7-days refeeding and fasting after EtOH withdrawal promoted mitochondria and lysosome function by restoring nuclear levels of transcription factor EB (TFEB)

**Fig. 8:**
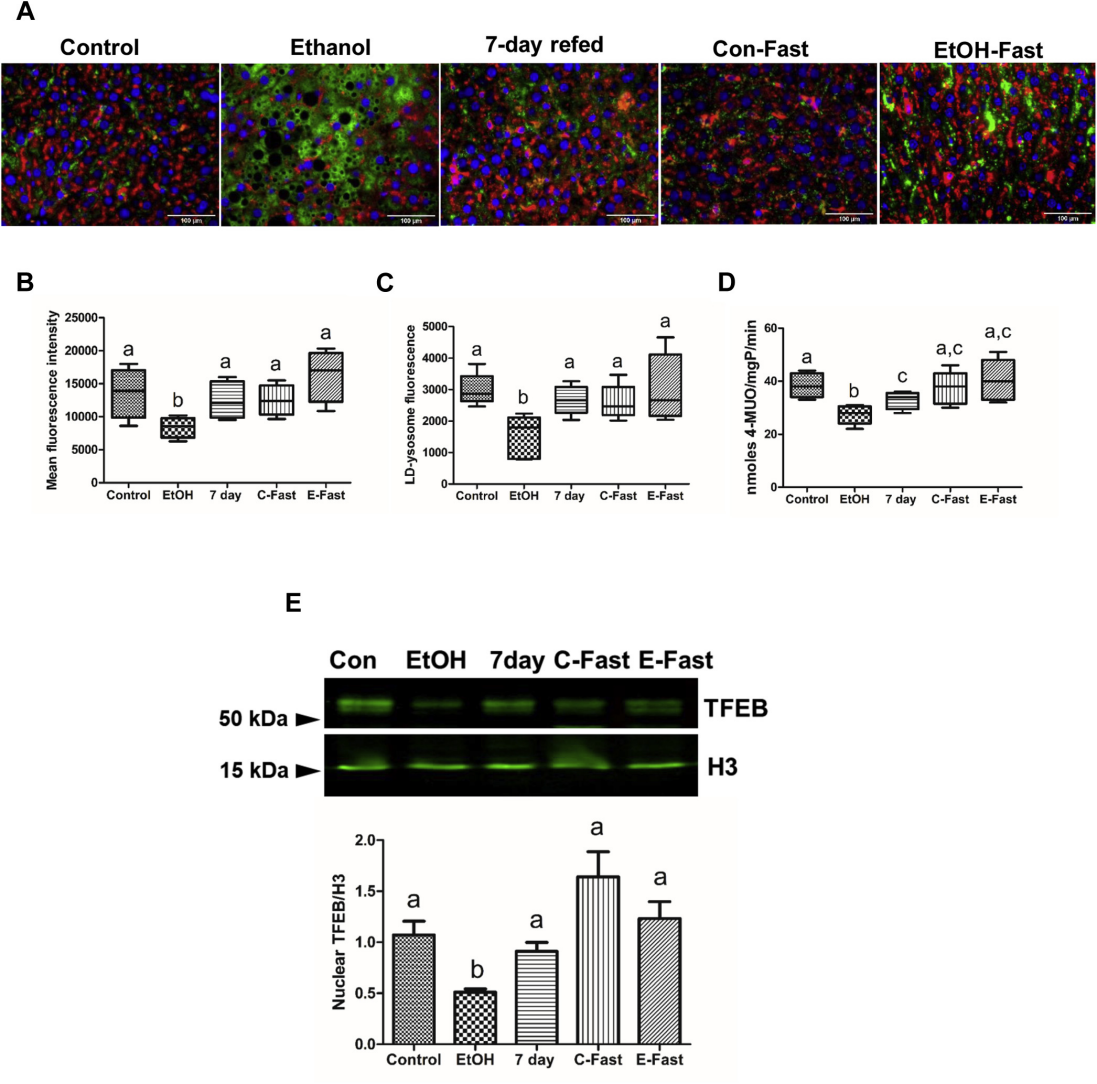
Refeeding and fasting after ethanol withdrawal promoted mitochondria and lysosomal function by restoring nuclear levels of transcription factor EB (TFEB). A: Immunostaining of liver sections with the antibody to lysosomal marker protein LAMP1 (red) and lipid droplet marker protein Plin-2 (green), from rats treated, as indicated. Fluorescence intensity quantification by ImageJ of (B) total lysosomal staining, (C) lysosomal staining around lipid droplets, and (D) specific activity of LAL in lysosomes isolated from livers of rats treated as indicated on the graph. E: TFEB in nuclear fractions. Data are the means ± SE of 6–8 animals/group. Bars with different letters are significantly different. Bars with the same letter are not significantly different, *P* ≤ 0.05. LAL, lysosomal acid lipase; LAMP, lysosome-associated membrane protein; PLIN, perilipin.

Using microscopic examination and assay of lysosomal acid lipase (LAL) activity in lysosome enriched fractions, we found that 7-days refeeding and EtOH-fast reversed the EtOH-elicited reduction in lysosome function (Figs. 2, 5E). Compared with controls, the livers of EtOH-fed rats exhibited significantly lower lysosome numbers, as judged by LAMP-1 staining around (Fig. 8A–C). Seven-day refeeding and ethanol fast, both after ethanol withdrawal, each restored total lysosomes and their staining around LDs (Fig. 8A–C). LAL activity in lysosome-enriched fractions from ethanol-fed rats was 1.5-fold lower than in fractions from controls (Fig. 8D). LAL activity in lysosome fractions from 7-day refed animals was significantly higher than those from ethanol-fed rats, but lower than control animals. LAL activities in control-fast and ethanol-fast lysosomal fractions were comparable to those of controls (Fig. 8D). In livers of ethanol-fed rats, the nuclear content of transcription factor EB (TFEB), which transcriptionally regulates genes that encode proteins of lysosome and mitochondrial biogenesis (30), was 2-fold lower than controls (Fig. 8E). Nuclear TFEB levels were restored to control levels in livers of 7-day refed and ethanol-fast rats.

## Discussion

Long perceived as inert fat-storing intracellular vesicles, LDs gained attention for their role in regulating energy homeostasis in healthy liver cells and their oversupply in unhealthy hepatocytes of rodents or humans with alcohol- or diet-induced fatty liver (31). Recent understanding of LD biology reveals that the lipid and protein compositions of LDs are highly dynamic. Both influence intrinsic LD metabolism and signaling properties, which ultimately link them to changes in other cellular organelles (6, 8, 18). Here, we used a rat model of chronic alcohol feeding, and we report changes in the hepatic LD membrane-associated proteome induced by alcohol (ethanol) administration, which influences LD membrane proteome remodeling after ethanol withdrawal and abstinence. Specifically, the ethanol diet was either replaced with control diet or the animals were fasted for 48 h. In short, ethanol feeding promoted fatty liver, whereas subsequent refeeding of the control diet or fasting either fully or partially resolved steatosis.

The ethanol-induced hepatic accumulation of LDs (Fig. 1A) was associated with elevated hepatic TGs and serum NEFAs in these animals (Fig. 1B, C). NEFAs are major contributors to hepatic TG accumulation because they are rapidly taken up by hepatocytes and esterified with glycerol, forming TGs (2). As seen here, NEFA levels were completely normalized by 7-day refeeding but not by ethanol fast. Fasting induces adipose tissue lipolysis, which mobilized fatty acids from adipose tissue (32) into the sera of control-fast and ethanol-fast rats (Fig. 1C). Despite ethanol withdrawal, fasting of these animals likely replenished serum NEFA levels, thereby mitigating the complete attenuation of fatty liver after ethanol withdrawal. Although fasting ethanol-fed rats did not decrease serum NEFA levels, both it and 7-day refeeding enriched the contents of LD-associated proteins that are critical for reducing lipid accumulation (Fig. 2), thereby alleviating ethanol-induced fatty liver (Fig. 1A, B). Such alleviation included reductions in the amounts of (1) LD PLINs 3 and 5, the structural proteins that fortify LDs to prevent their breakdown (23); (2) hydroxysteroid proteins HSD17β13 and 11, both enzymes are involved in cholesterol and fatty acid metabolism and promote fatty liver in humans and animals (20, 21), and (3) CIDEB of the CIDE family of proteins, which promotes LD fusion to create larger LDs (18). In addition, fasting (ethanol fast) and 7-day refeeding after ethanol withdrawal both restored (1) the active forms of HSL and ATGL, lipases which break down larger LDs to smaller LDs, thereby facilitating lipophagy (33) and (2) LAMP1 and LAMP2A levels, both indicating enhanced interaction of lysosomes with LDs, thereby enhancing macrolipophagy and chaperone-mediated autophagy. Differential regulation/expression of these latter proteins on the surfaces of LDs reflects the degree of fatty liver after subjecting rats to the aforementioned treatments. These data lead us to suggest that LD membrane-associated proteins participate directly in regulating LD size and LD turnover (synthesis and degradation).

GO analyses of LD proteins from pair-fed control rats, identified by MS, revealed that LD membrane proteins' BFs are largely grouped under sterol and fatty acid biosynthetic pathways, fatty acid beta oxidation, electron transport, oxidative phosphorylation, oxidoreductase activity, and vesicle trafficking. Moreover, a subset of these latter proteins was identified as proteins of the mitochondrion, ER, peroxisome, and Golgi apparatus (Fig. 3B). These data confirm widespread microscopic data that LDs interact by direct contact with other cellular organelles. Electron microscope studies (34) provide visual evidence of such contacts, which are facilitated by protein-based interorganelle contact sites on LDs that allow trafficking-independent communication with other organelles to regulate LD dynamics (6). These data indicate that LD metabolism and turnover depends on (1) lipid-regulating proteins that are tightly associated with LDs and (2) frequent communication with other organelles. Here, we have shown clear evidence that proteins associated with other organelles and which participate in lipid metabolism by direct interaction with LDs were detected on isolated LDs from livers of control and ethanol-fed animals.

Chronic ethanol feeding altered 338 different LD membrane-associated proteins and induced drastic changes in the LD proteome network (Fig. 4). Here, our proteomic analyses identified additional proteins associated with affected BFs, as judged by WB analyses of LD membrane proteins (Fig. 2). Proteomic quantifications confirmed our WB data that ethanol feeding elevated the levels of PLIN-3, PLIN-5, CIDEB, and HSD17β13 (Fig. 4B). Proteomics also revealed that ethanol administration increased the levels of LSS, squalene monooxygenase, 3-keto-steroid reductase (HSD17β7), and several cytochrome P450 isozymes that catalyze steroid and cholesterol biosynthesis. These data confirm our WB quantifications (Fig. 2) of steroidogenic proteins (HSD17β11/13) that promote fatty liver. WBs also revealed that chronic ethanol feeding decreased the level of pATGL, while it also decreased LAMP1, a reliable index of the lysosome content (Fig. 2). Proteomics of LDs from ethanol-fed rats confirmed these findings by revealing higher levels of additional ATGL inhibitors (G0s2 and Fas-associated factor family member 2) and lower levels of other lysosomal proteins, including lysosomal integral membrane protein 2 and ATP6V0A1. IPAs of all the significantly altered proteins revealed that mitochondrial pathways were predominantly downregulated in LDs of ethanol-fed rats (Fig. 4C, D), whereas steroid and cholesterol biosynthetic pathways were the most highly activated (Fig. 4C). These findings are well aligned with previous results (9, 10, 11, 35, 36) that ethanol feeding increases hepatic steroid and cholesterol contents while it downregulates lipases and lysosome contents thereby slowing LD breakdown via lipolysis and lipophagy.

Refeeding and fasting ethanol-fed rats each had contrasting effects on the LD membrane proteome. Compared with pair-fed control rats, 7-day refeeding after ethanol withdrawal altered 93 proteins (Fig. 5B) which clustered rather closely with controls on the PCA plot (Fig. 5A), indicating that ethanol-induced changes were reversed by refeeding (Fig. 5A). However, ethanol fast altered 448 LD proteins and these remained very distinct from LDS of pair-fed control, ethanol-fed and 7-day refed animals (Fig. 5A). These findings lead us to suggest that ethanol fast operates through a distinct mechanism to remodel ethanol-induced changes in the LD membrane proteome. Upon analyzing more proteins, in addition to those described above that participate in lipogenesis and lipid catabolism, we found that ethanol feeding induced different proteins that accelerate steroid hormone and cholesterol biosynthesis (Fig. 5E). These include cytochrome b5 reductase 3, DHCR7, sterol-4-alpha-carboxylate 3-dehydrogenase, decarboxylating, TM7SF2, and DHCR7 that stimulate steroid and cholesterol biosynthesis and METTL7A, and ACSM1 and ACSM5 that enhance fatty acid activation and LD formation. Elevations of these proteins occurred simultaneously with decreased levels of proteins that enhance lysosomal activity (ATP6V0A1 and SCARB1). The latter ethanol-induced changes by were completely reversed by 7-day refeeding with the control diet. Although ethanol fast enhanced the levels of ACSM1 and ACSM5, it largely attenuated or normalized the proteins altered by ethanol, but it markedly elevated lysosomal proteins. IPAs of canonical pathways (Fig. 6) and manual review of mitochondrial proteins (Supplemental Table S8) indicated that both 7-day refeeding and ethanol fast reversed the ethanol-elicited enhancement of cholesterol biosynthesis and increased the levels of mitochondrial proteins (Fig. 6B, D). Review of BF categories also supported our canonical pathway analyses. BF categories in 7-day refed and ethanol-fasted animals, compared with controls, revealed that there was no predictable activation state in all the biological categories in 7-day refed rats, indicating that the effects of refeeding were not significantly different and were therefore equivalent to those from controls. However, ethanol fast caused a 2-fold decrease in the LD levels of ethanol-induced proteins that promote lipogenesis (Table 2, Fig. 7A, B). Ethanol fast also enhanced the levels of LD proteins that participate in lipid oxidation in peroxisomes and mitochondria, and it enhanced the levels of Rab and other vesicle-trafficking proteins, which facilitate LD interaction with other organelles and LD degradation in lysosomes (Table 2). These findings indicate that, although 7-day refeeding and ethanol fast distinctly remodeled the ethanol-induced LD proteome, they both attenuated steroid and cholesterol biosynthesis, restored mitochondrial proteins, and promoted lipolysis and lipophagy, both pathways of LD degradation.

Microscopic analyses and catalytic activity measurements of mitochondria and lysosomes further confirmed that 7-day refeeding and ethanol fast restored the activities of these organelles to enhance lipid breakdown (Fig. 7E, F and Fig. 8A, B). Staining of both mitochondria and lysosomes was lower in livers of ethanol-fed rats. Staining intensity increased after both 7-day refeeding and ethanol fast. The latter two interventions not only restored normal levels of mitochondrial and lysosome makers but also normalized mitochondrial complex I activity and the levels of LAL, both of which declined after ethanol administration (Fig. 8D). Restoration of mitochondrial and lysosomal function correlated positively with restored nuclear levels of TFEB, which regulates autophagy, by enhancing the biogenesis of lysosomes and mitochondria (30) (Fig. 8E). Although ethanol administration decreased the levels of nuclear TFEB, both 7-day refeeding and ethanol fast of ethanol-fed rats restored its levels in rat liver nuclei. These data lead us to suggest that 7-day refeeding and ethanol fast restored nuclear TFEB, which, in turn, reactivated mitochondrial and lysosomal functions. All these findings suggest that, refeeding or fasting after ethanol withdrawal attenuated LD accumulation by reducing the levels of LD membrane proteins that promote lipid biosynthesis, while simultaneously promoting those that breakdown LDs and oxidize the lipids within, in lysosomes and mitochondria, respectively.

In summary, chronic ethanol administration significantly altered the LD membrane-associated proteome, enriching lipid biosynthetic proteins and reducing lipid degrading proteins, thereby conferring LD resistance to breakdown. These ethanol-elicited changes were further associated with lower levels of mitochondrial and lysosomal proteins on the LD surface. These findings indicate reduced contents (and activities) of these organelles in liver cells of ethanol-fed rats. Our findings also lead us to suggest that ethanol feeding likely disrupts the direct communication of LDs with mitochondria and lysosomes because the biogenesis of each is reduced. The latter events exacerbate fatty liver by slowing LD catabolism. Seven-day refeeding or fasting of ethanol-fed rats, each distinctly remodeled the ethanol-modified LD proteome by lowering lipid biosynthetic proteins and restoring LD catabolic proteins, specifically those from mitochondria and lysosomes, thereby alleviating ethanol-induced fatty liver. Although both refeeding and fasting after ethanol withdrawal attenuated ethanol-induced fatty liver, refeeding the control diet clearly reduced unexpected complications that arose from fasting. One of these included higher NEFA levels that caused a slower decline in hepatic fat, which was potentially hepatotoxic. In conclusion, our data clearly support the notion that alcohol withdrawal, along with adequate nutritional support, is necessary for managing alcohol-induced liver injury.

## Data availability

All data are contained within the article or in the supplemental material section. Raw mass spectrometry data are available at MassIVE public repository site [MassIVE Dataset Summary (ucsd.edu)].

## Supplemental data

This article contains supplemental data.

## Conflict of interest

The authors declare that they have no conflicts of interest with the contents of this article.
