# Supplementary material for: Lipid droplet membrane proteome remodeling parallels ethanol-induced hepatic steatosis and its resolution
Source: J Lipid Res. 2021 Feb 20;62:100049. doi: 10.1016/j.jlr.2021.100049 (PMC8010705; doi:10.1016/j.jlr.2021.100049)
Supplement: Supplementary file 1 — Supplemental Figures S1 and S2 [file mmc1.pdf]

Supplementary Fig 1.

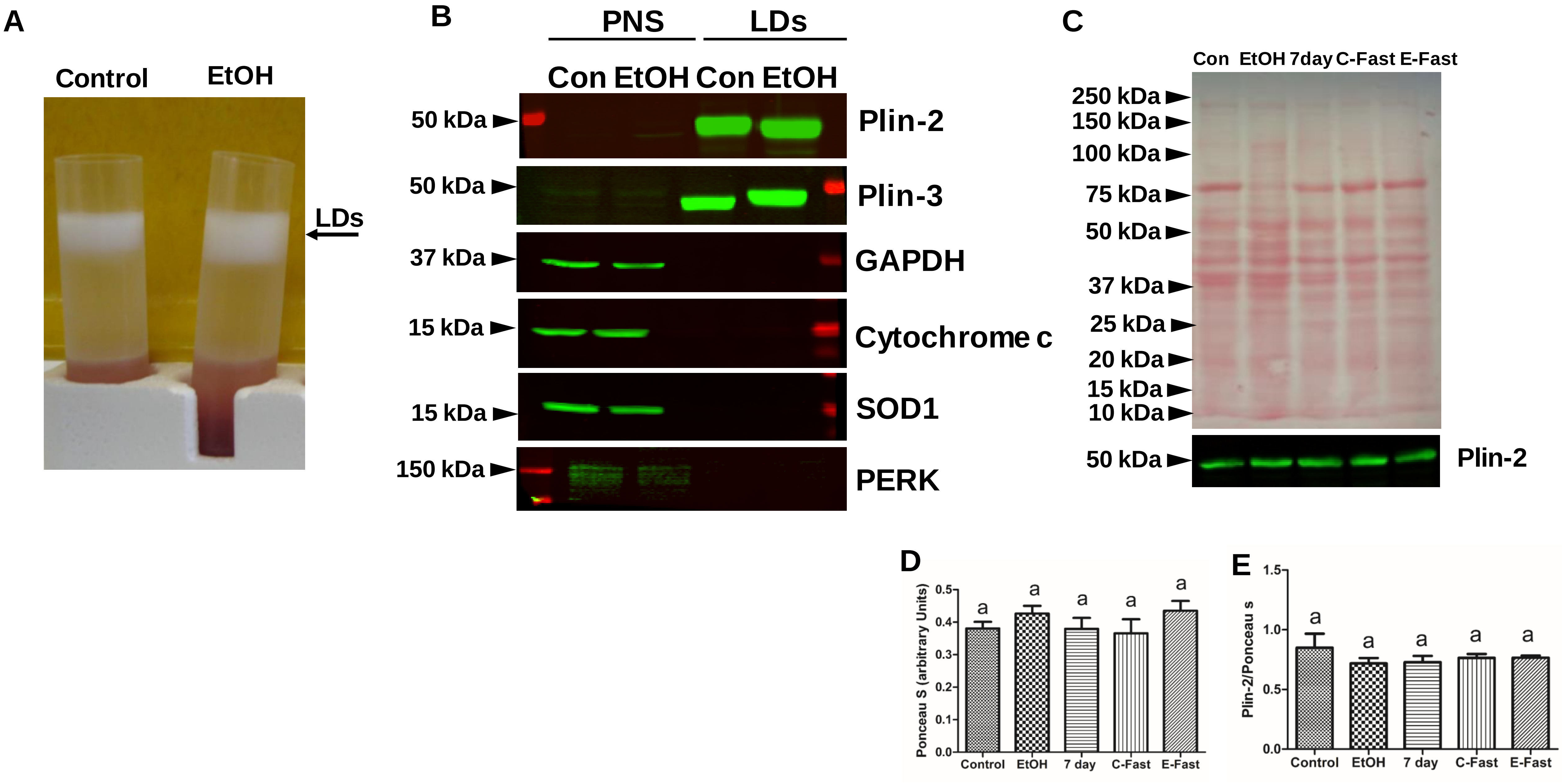

S Fig 1. Purification and quantification of lipid droplet membrane proteins. **(A)** Representative image showing buoyant lipid droplets (LDs) after gradient centrifugation of post nuclear supernatants (PNS) obtained from the livers of control and ethanol-fed rats **(B)** Representative Western blot show absence of common contaminating proteins such as cytosol (GAPDH), mitochondria (Cytochrome C and Super Oxide dismutase (SOD1) and endoplasmic reticulum (protein kinase RNA-like endoplasmic reticulum kinase (PERK)) in LDs isolated from control and ethanol-fed groups **(C)** ponceau S staining and Western blotting show protein bands and PLIN-2 levels, respectively, in equal protein loaded LD fractions from different groups **(D)** densitometry quantification of ponceau S staining and **(E)** PLIN-2 levels after normalization with ponceau S staining in each group. Data are mean values of ± S.E.M. of 4 to 6 animals per group. Bars sharing different letters are significantly different. Bars sharing the same letter are not significantly different, $P \leq 0.05$.

Supplementary Fig 2.

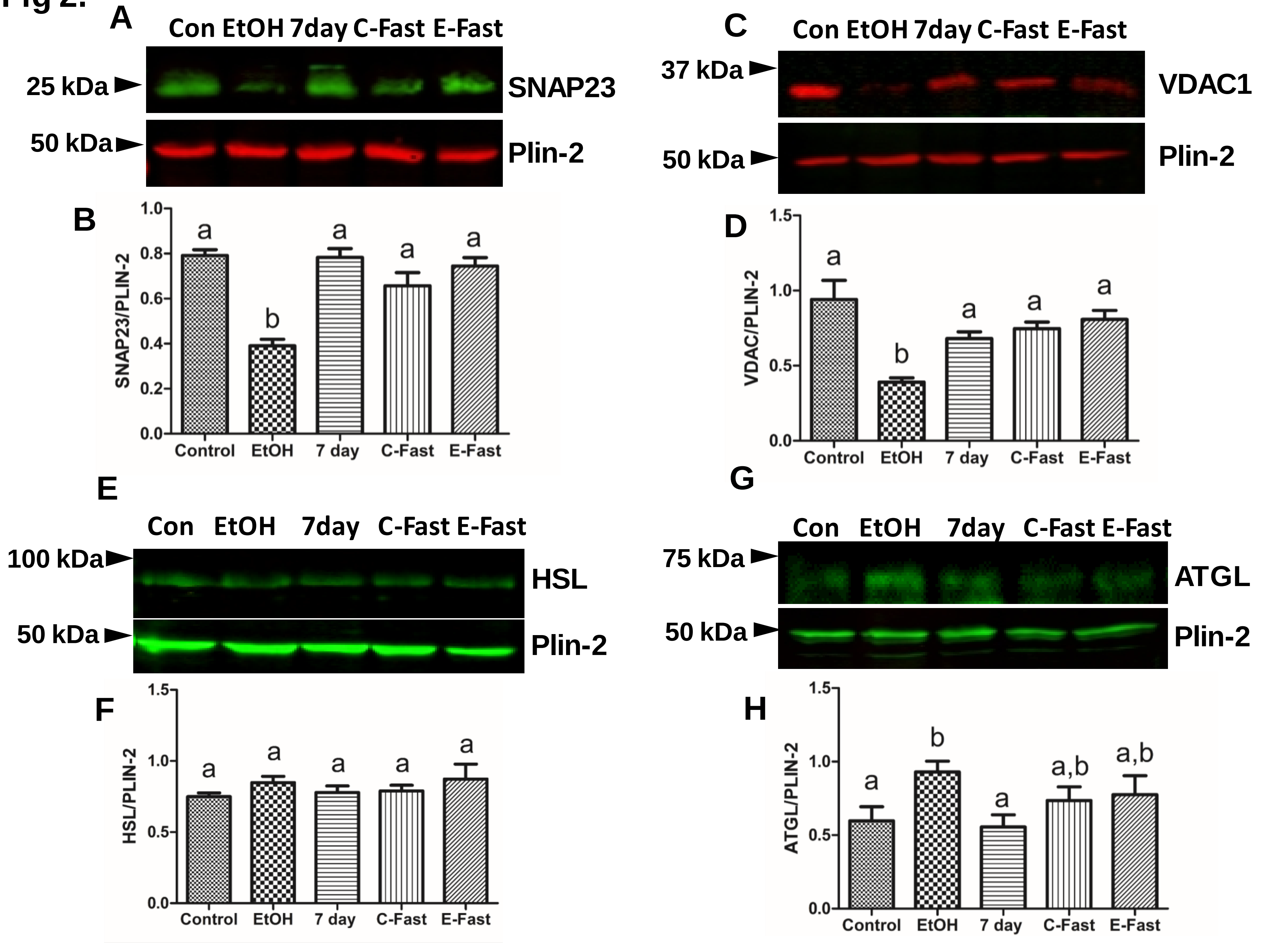

S Fig 2. Expression of select lipid droplet membrane proteins. **(A)** Representative Western blot and densitometry quantification of **(A&B)** Synaptosomal-associated protein 23 (SNAP23) **(C&D)** Voltage-dependent anion channel (VDAC1) (**E&F)** Hormone sensitive lipase (HSL) and **(G&H)** Adipose tissue triglyceride lipase (ATGL) in LDs from different experimental groups. Data are mean values of ± S.E.M. of 4 to 6 animals per group. Bars sharing different letters are significantly different. Bars sharing the same letter are not significantly different, $P \leq 0.05$.
